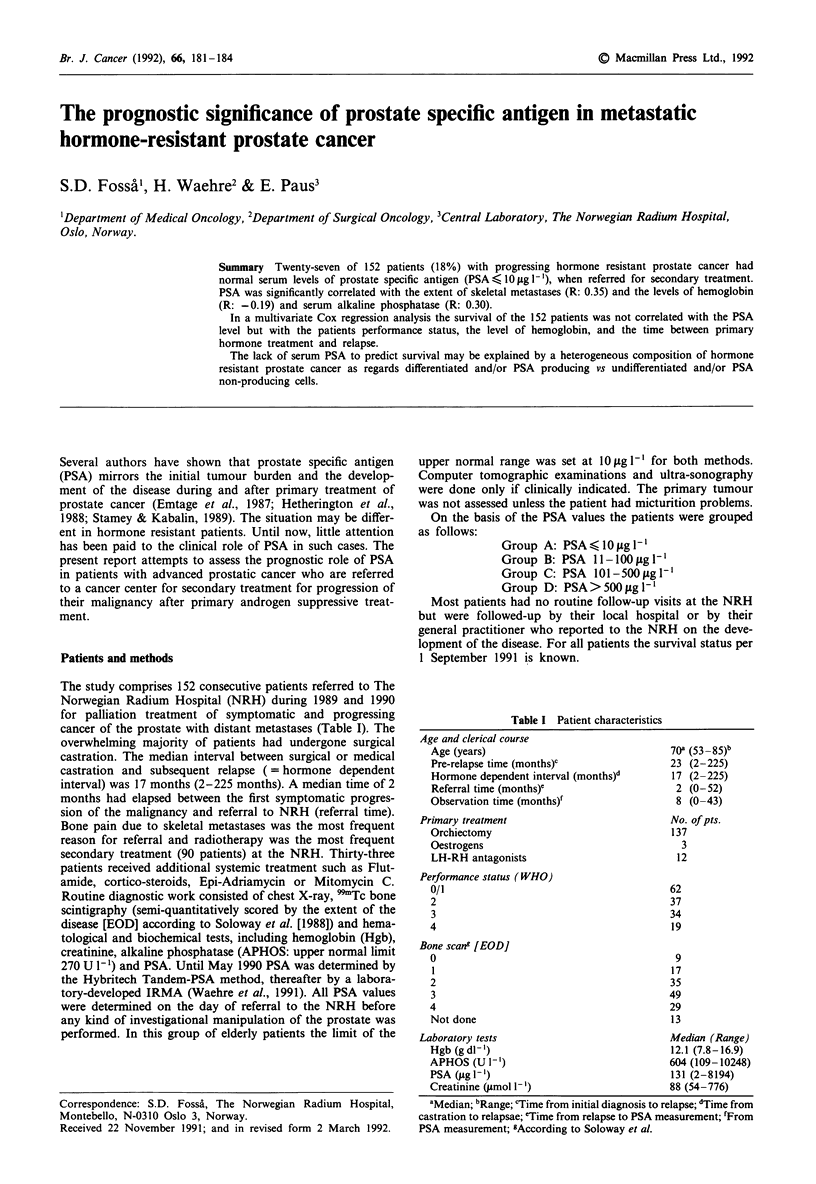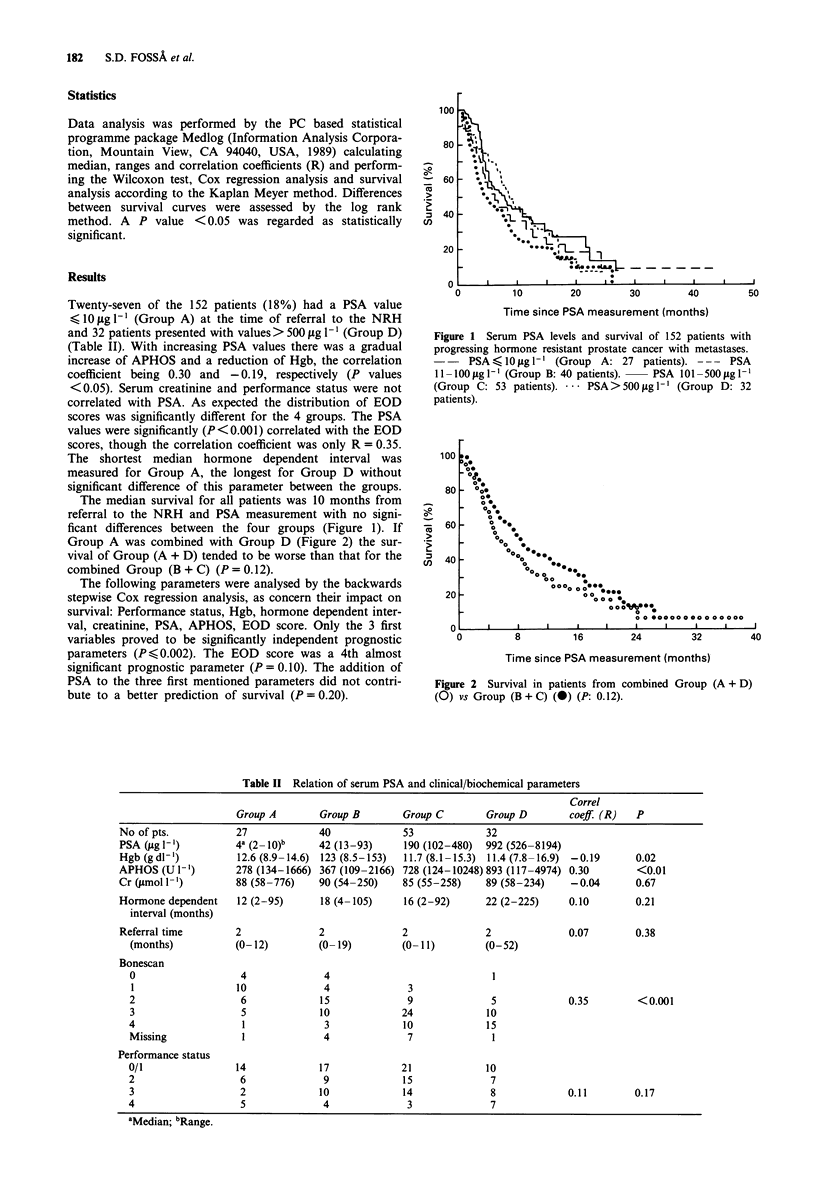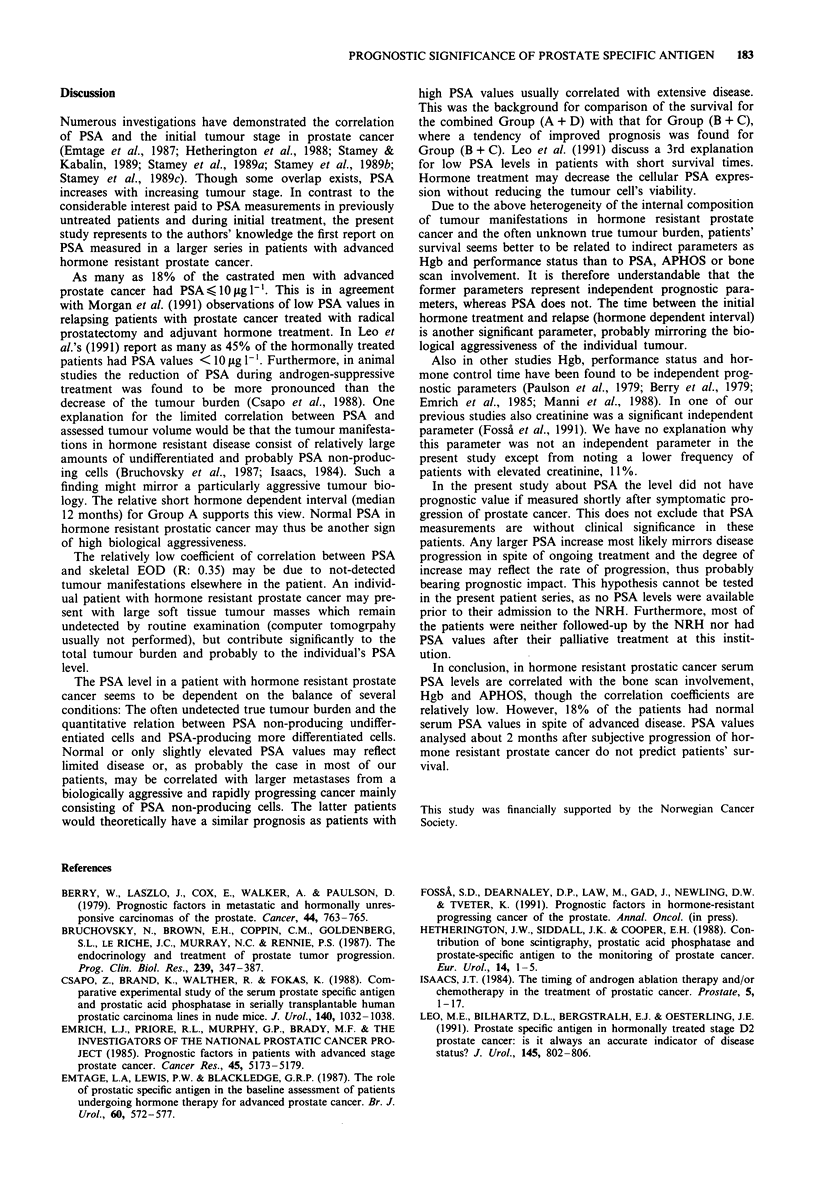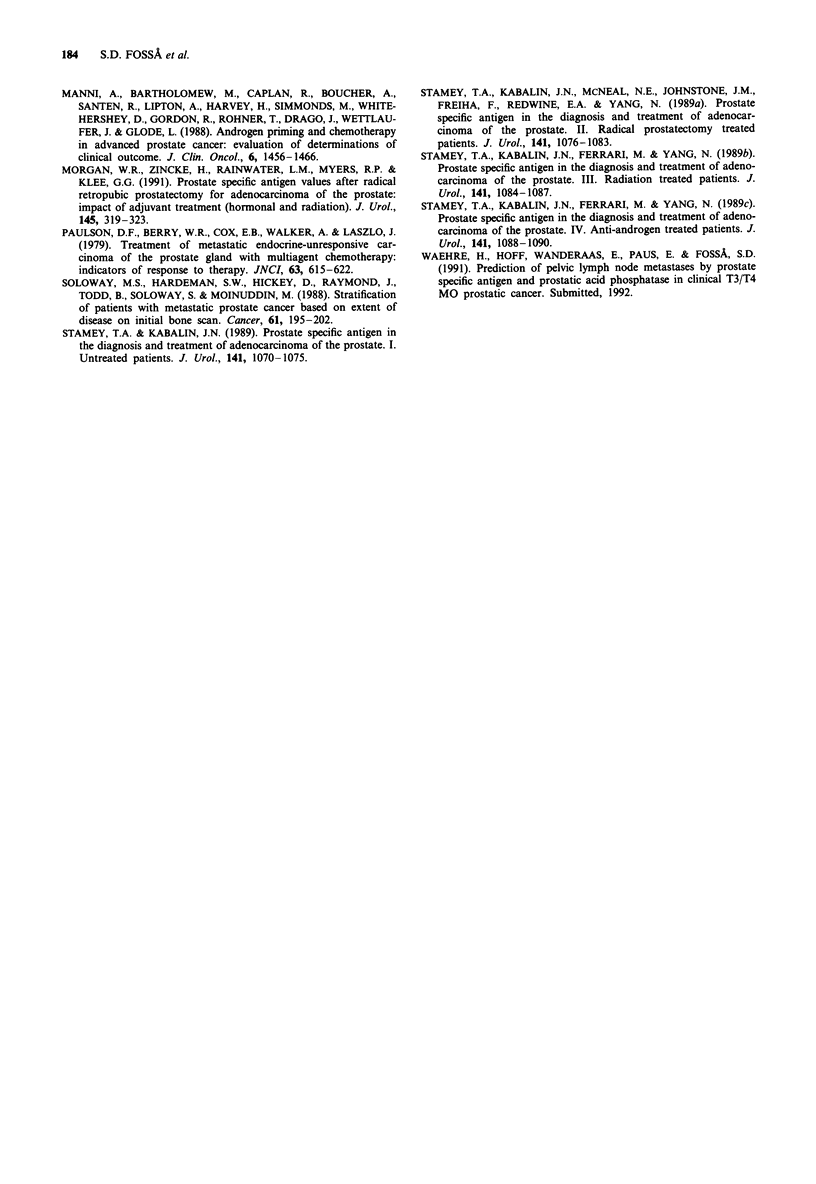# The prognostic significance of prostate specific antigen in metastatic hormone-resistant prostate cancer.

**DOI:** 10.1038/bjc.1992.239

**Published:** 1992-07

**Authors:** S. D. Fosså, H. Waehre, E. Paus

**Affiliations:** Department of Medical Oncology, Norwegian Radium Hospital, Oslo.

## Abstract

Twenty-seven of 152 patients (18%) with progressing hormone resistant prostate cancer had normal serum levels of prostate specific antigen (PSA less than or equal to 10 micrograms l-1), when referred for secondary treatment. PSA was significantly correlated with the extent of skeletal metastases (R: 0.35) and the levels of hemoglobin (R: -0.19) and serum alkaline phosphatase (R: 0.30). In a multivariate Cox regression analysis the survival of the 152 patients was not correlated with the PSA level but with the patients performance status, the level of hemoglobin, and the time between primary hormone treatment and relapse. The lack of serum PSA to predict survival may be explained by a heterogenous composition of hormone resistant prostate cancer as regards differentiated and/or PSA producing vs undifferentiated and/or PSA non-producing cells.


Br. J. Cancer (1992), 66, 181–184 © Macmillan Press Ltd., 1992

### The prognostic significance of prostate specific antigen in metastatic hormone-resistant prostate cancer

S.D. Fosså[1], H. Waehre[2] & E. Paus[3]

[1]*Department of Medical Oncology,* [2]*Department of Surgical Oncology,* [3]*Central Laboratory, The Norwegian Radium Hospital, Oslo, Norway.*

**Summary** Twenty-seven of 152 patients (18%) with progressing hormone resistant prostate cancer had normal serum levels of prostate specific antigen (PSA $\leqslant 10\,\mu g\,l^{-1}$), when referred for secondary treatment. PSA was significantly correlated with the extent of skeletal metastases (R: 0.35) and the levels of hemoglobin (R: −0.19) and serum alkaline phosphatase (R: 0.30).

In a multivariate Cox regression analysis the survival of the 152 patients was not correlated with the PSA level but with the patients performance status, the level of hemoglobin, and the time between primary hormone treatment and relapse.

The lack of serum PSA to predict survival may be explained by a heterogeneous composition of hormone resistant prostate cancer as regards differentiated and/or PSA producing *vs* undifferentiated and/or PSA non-producing cells.

Several authors have shown that prostate specific antigen (PSA) mirrors the initial tumour burden and the development of the disease during and after primary treatment of prostate cancer (Emtage *et al.*, 1987; Hetherington *et al.*, 1988; Stamey & Kabalin, 1989). The situation may be different in hormone resistant patients. Until now, little attention has been paid to the clinical role of PSA in such cases. The present report attempts to assess the prognostic role of PSA in patients with advanced prostatic cancer who are referred to a cancer center for secondary treatment for progression of their malignancy after primary androgen suppressive treatment.

### Patients and methods

The study comprises 152 consecutive patients referred to The Norwegian Radium Hospital (NRH) during 1989 and 1990 for palliation treatment of symptomatic and progressing cancer of the prostate with distant metastases (Table I). The overwhelming majority of patients had undergone surgical castration. The median interval between surgical or medical castration and subsequent relapse ( = hormone dependent interval) was 17 months (2–225 months). A median time of 2 months had elapsed between the first symptomatic progression of the malignancy and referral to NRH (referral time). Bone pain due to skeletal metastases was the most frequent reason for referral and radiotherapy was the most frequent secondary treatment (90 patients) at the NRH. Thirty-three patients received additional systemic treatment such as Flutamide, cortico-steroids, Epi-Adriamycin or Mitomycin C. Routine diagnostic work consisted of chest X-ray, $^{99m}Tc$ bone scintigraphy (semi-quantitatively scored by the extent of the disease [EOD] according to Soloway *et al.* [1988]) and hematological and biochemical tests, including hemoglobin (Hgb), creatinine, alkaline phosphatase (APHOS: upper normal limit $270\,U\,l^{-1}$) and PSA. Until May 1990 PSA was determined by the Hybritech Tandem-PSA method, thereafter by a laboratory-developed IRMA (Waehre *et al.*, 1991). All PSA values were determined on the day of referral to the NRH before any kind of investigational manipulation of the prostate was performed. In this group of elderly patients the limit of the upper normal range was set at $10\,\mu g\,l^{-1}$ for both methods. Computer tomographic examinations and ultra-sonography were done only if clinically indicated. The primary tumour was not assessed unless the patient had micturition problems.

On the basis of the PSA values the patients were grouped as follows:

Group A: PSA $\leqslant 10\,\mu g\,l^{-1}$

Group B: PSA 11–100 $\mu g\,l^{-1}$

Group C: PSA 101–500 $\mu g\,l^{-1}$

Group D: PSA $> 500\,\mu g\,l^{-1}$

Most patients had no routine follow-up visits at the NRH but were followed-up by their local hospital or by their general practitioner who reported to the NRH on the development of the disease. For all patients the survival status per 1 September 1991 is known.

**Table I** Patient characteristics

| | |
|---|---|
| *Age and clerical course* | |
| Age (years) | 70[a] (53–85)[b] |
| Pre-relapse time (months)[c] | 23 (2–225) |
| Hormone dependent interval (months)[d] | 17 (2–225) |
| Referral time (months)[e] | 2 (0–52) |
| Observation time (months)[f] | 8 (0–43) |
| *Primary treatment* | *No. of pts.* |
| Orchiectomy | 137 |
| Oestrogens | 3 |
| LH-RH antagonists | 12 |
| *Performance status (WHO)* | |
| 0/1 | 62 |
| 2 | 37 |
| 3 | 34 |
| 4 | 19 |
| *Bone scan[g] [EOD]* | |
| 0 | 9 |
| 1 | 17 |
| 2 | 35 |
| 3 | 49 |
| 4 | 29 |
| Not done | 13 |
| *Laboratory tests* | *Median (Range)* |
| Hgb ($g\,dl^{-1}$) | 12.1 (7.8–16.9) |
| APHOS ($U\,l^{-1}$) | 604 (109–10248) |
| PSA ($\mu g\,l^{-1}$) | 131 (2–8194) |
| Creatinine ($\mu mol\,l^{-1}$) | 88 (54–776) |

[a]Median; [b]Range; [c]Time from initial diagnosis to relapse; [d]Time from castration to relapsae; [e]Time from relapse to PSA measurement; [f]From PSA measurement; [g]According to Soloway *et al.*

Correspondence: S.D. Fosså, The Norwegian Radium Hospital, Montebello, N-0310 Oslo 3, Norway.
Received 22 November 1991; and in revised form 2 March 1992.

### Statistics

Data analysis was performed by the PC based statistical programme package Medlog (Information Analysis Corporation, Mountain View, CA 94040, USA, 1989) calculating median, ranges and correlation coefficients (R) and performing the Wilcoxon test, Cox regression analysis and survival analysis according to the Kaplan Meyer method. Differences between survival curves were assessed by the log rank method. A $P$ value $<0.05$ was regarded as statistically significant.

### Results

Twenty-seven of the 152 patients (18%) had a PSA value $\leqslant 10\ \mu g\ l^{-1}$ (Group A) at the time of referral to the NRH and 32 patients presented with values $> 500\ \mu g\ l^{-1}$ (Group D) (Table II). With increasing PSA values there was a gradual increase of APHOS and a reduction of Hgb, the correlation coefficient being 0.30 and −0.19, respectively ($P$ values $<0.05$). Serum creatinine and performance status were not correlated with PSA. As expected the distribution of EOD scores was significantly different for the 4 groups. The PSA values were significantly ($P<0.001$) correlated with the EOD scores, though the correlation coefficient was only $R = 0.35$. The shortest median hormone dependent interval was measured for Group A, the longest for Group D without significant difference of this parameter between the groups.

The median survival for all patients was 10 months from referral to the NRH and PSA measurement with no significant differences between the four groups (Figure 1). If Group A was combined with Group D (Figure 2) the survival of Group (A + D) tended to be worse than that for the combined Group (B + C) ($P = 0.12$).

The following parameters were analysed by the backwards stepwise Cox regression analysis, as concern their impact on survival: Performance status, Hgb, hormone dependent interval, creatinine, PSA, APHOS, EOD score. Only the 3 first variables proved to be significantly independent prognostic parameters ($P \leqslant 0.002$). The EOD score was a 4th almost significant prognostic parameter ($P = 0.10$). The addition of PSA to the three first mentioned parameters did not contribute to a better prediction of survival ($P = 0.20$).

**Figure 1** Serum PSA levels and survival of 152 patients with progressing hormone resistant prostate cancer with metastases. — — PSA $\leqslant 10\ \mu g\ l^{-1}$ (Group A: 27 patients). - - - PSA 11–100 $\mu g\ l^{-1}$ (Group B: 40 patients). —— PSA 101–500 $\mu g\ l^{-1}$ (Group C: 53 patients). ··· PSA $> 500\ \mu g\ l^{-1}$ (Group D: 32 patients).

**Figure 2** Survival in patients from combined Group (A + D) (○) *vs* Group (B + C) (●) ($P$: 0.12).

**Table II** Relation of serum PSA and clinical/biochemical parameters

| | *Group A* | *Group B* | *Group C* | *Group D* | *Correl coeff. (R)* | *P* |
|---|---|---|---|---|---|---|
| No of pts. | 27 | 40 | 53 | 32 | | |
| PSA ($\mu g\ l^{-1}$) | 4[a] (2–10)[b] | 42 (13–93) | 190 (102–480) | 992 (526–8194) | | |
| Hgb ($g\ dl^{-1}$) | 12.6 (8.9–14.6) | 123 (8.5–153) | 11.7 (8.1–15.3) | 11.4 (7.8–16.9) | −0.19 | 0.02 |
| APHOS ($U\ l^{-1}$) | 278 (134–1666) | 367 (109–2166) | 728 (124–10248) | 893 (117–4974) | 0.30 | <0.01 |
| Cr ($\mu mol\ l^{-1}$) | 88 (58–776) | 90 (54–250) | 85 (55–258) | 89 (58–234) | −0.04 | 0.67 |
| Hormone dependent interval (months) | 12 (2–95) | 18 (4–105) | 16 (2–92) | 22 (2–225) | 0.10 | 0.21 |
| Referral time (months) | 2 (0–12) | 2 (0–19) | 2 (0–11) | 2 (0–52) | 0.07 | 0.38 |
| Bonescan | | | | | | |
| 0 | 4 | 4 | | 1 | | |
| 1 | 10 | 4 | 3 | | | |
| 2 | 6 | 15 | 9 | 5 | 0.35 | <0.001 |
| 3 | 5 | 10 | 24 | 10 | | |
| 4 | 1 | 3 | 10 | 15 | | |
| Missing | 1 | 4 | 7 | 1 | | |
| Performance status | | | | | | |
| 0/1 | 14 | 17 | 21 | 10 | | |
| 2 | 6 | 9 | 15 | 7 | | |
| 3 | 2 | 10 | 14 | 8 | 0.11 | 0.17 |
| 4 | 5 | 4 | 3 | 7 | | |

[a]Median; [b]Range.

### Discussion

Numerous investigations have demonstrated the correlation of PSA and the initial tumour stage in prostate cancer (Emtage *et al.*, 1987; Hetherington *et al.*, 1988; Stamey & Kabalin, 1989; Stamey *et al.*, 1989*a*; Stamey *et al.*, 1989*b*; Stamey *et al.*, 1989*c*). Though some overlap exists, PSA increases with increasing tumour stage. In contrast to the considerable interest paid to PSA measurements in previously untreated patients and during initial treatment, the present study represents to the authors' knowledge the first report on PSA measured in a larger series in patients with advanced hormone resistant prostate cancer.

As many as 18% of the castrated men with advanced prostate cancer had PSA $\leqslant 10\,\mu g\,l^{-1}$. This is in agreement with Morgan *et al.* (1991) observations of low PSA values in relapsing patients with prostate cancer treated with radical prostatectomy and adjuvant hormone treatment. In Leo *et al.*'s (1991) report as many as 45% of the hormonally treated patients had PSA values $< 10\,\mu g\,l^{-1}$. Furthermore, in animal studies the reduction of PSA during androgen-suppressive treatment was found to be more pronounced than the decrease of the tumour burden (Csapo *et al.*, 1988). One explanation for the limited correlation between PSA and assessed tumour volume would be that the tumour manifestations in hormone resistant disease consist of relatively large amounts of undifferentiated and probably PSA non-producing cells (Bruchovsky *et al.*, 1987; Isaacs, 1984). Such a finding might mirror a particularly aggressive tumour biology. The relative short hormone dependent interval (median 12 months) for Group A supports this view. Normal PSA in hormone resistant prostatic cancer may thus be another sign of high biological aggressiveness.

The relatively low coefficient of correlation between PSA and skeletal EOD (R: 0.35) may be due to not-detected tumour manifestations elsewhere in the patient. An individual patient with hormone resistant prostate cancer may present with large soft tissue tumour masses which remain undetected by routine examination (computer tomogrpahy usually not performed), but contribute significantly to the total tumour burden and probably to the individual's PSA level.

The PSA level in a patient with hormone resistant prostate cancer seems to be dependent on the balance of several conditions: The often undetected true tumour burden and the quantitative relation between PSA non-producing undifferentiated cells and PSA-producing more differentiated cells. Normal or only slightly elevated PSA values may reflect limited disease or, as probably the case in most of our patients, may be correlated with larger metastases from a biologically aggressive and rapidly progressing cancer mainly consisting of PSA non-producing cells. The latter patients would theoretically have a similar prognosis as patients with high PSA values usually correlated with extensive disease. This was the background for comparison of the survival for the combined Group (A + D) with that for Group (B + C), where a tendency of improved prognosis was found for Group (B + C). Leo *et al.* (1991) discuss a 3rd explanation for low PSA levels in patients with short survival times. Hormone treatment may decrease the cellular PSA expression without reducing the tumour cell's viability.

Due to the above heterogeneity of the internal composition of tumour manifestations in hormone resistant prostate cancer and the often unknown true tumour burden, patients' survival seems better to be related to indirect parameters as Hgb and performance status than to PSA, APHOS or bone scan involvement. It is therefore understandable that the former parameters represent independent prognostic parameters, whereas PSA does not. The time between the initial hormone treatment and relapse (hormone dependent interval) is another significant parameter, probably mirroring the biological aggressiveness of the individual tumour.

Also in other studies Hgb, performance status and hormone control time have been found to be independent prognostic parameters (Paulson *et al.*, 1979; Berry *et al.*, 1979; Emrich *et al.*, 1985; Manni *et al.*, 1988). In one of our previous studies also creatinine was a significant independent parameter (Fosså *et al.*, 1991). We have no explanation why this parameter was not an independent parameter in the present study except from noting a lower frequency of patients with elevated creatinine, 11%.

In the present study about PSA the level did not have prognostic value if measured shortly after symptomatic progression of prostate cancer. This does not exclude that PSA measurements are without clinical significance in these patients. Any larger PSA increase most likely mirrors disease progression in spite of ongoing treatment and the degree of increase may reflect the rate of progression, thus probably bearing prognostic impact. This hypothesis cannot be tested in the present patient series, as no PSA levels were available prior to their admission to the NRH. Furthermore, most of the patients were neither followed-up by the NRH nor had PSA values after their palliative treatment at this institution.

In conclusion, in hormone resistant prostatic cancer serum PSA levels are correlated with the bone scan involvement, Hgb and APHOS, though the correlation coefficients are relatively low. However, 18% of the patients had normal serum PSA values in spite of advanced disease. PSA values analysed about 2 months after subjective progression of hormone resistant prostate cancer do not predict patients' survival.

This study was financially supported by the Norwegian Cancer Society.

### References

BERRY, W., LASZLO, J., COX, E., WALKER, A. & PAULSON, D. (1979). Prognostic factors in metastatic and hormonally unresponsive carcinomas of the prostate. *Cancer*, **44**, 763–765.

BRUCHOVSKY, N., BROWN, E.H., COPPIN, C.M., GOLDENBERG, S.L., LE RICHE, J.C., MURRAY, N.C. & RENNIE, P.S. (1987). The endocrinology and treatment of prostate tumor progression. *Prog. Clin. Biol. Res.*, **239**, 347–387.

CSAPO, Z., BRAND, K., WALTHER, R. & FOKAS, K. (1988). Comparative experimental study of the serum prostate specific antigen and prostatic acid phosphatase in serially transplantable human prostatic carcinoma lines in nude mice. *J. Urol.*, **140**, 1032–1038.

EMRICH, L.J., PRIORE, R.L., MURPHY, G.P., BRADY, M.F. & THE INVESTIGATORS OF THE NATIONAL PROSTATIC CANCER PROJECT (1985). Prognostic factors in patients with advanced stage prostate cancer. *Cancer Res.*, **45**, 5173–5179.

EMTAGE, L.A, LEWIS, P.W. & BLACKLEDGE, G.R.P. (1987). The role of prostatic specific antigen in the baseline assessment of patients undergoing hormone therapy for advanced prostate cancer. *Br. J. Urol.*, **60**, 572–577.

FOSSÅ, S.D., DEARNALEY, D.P., LAW, M., GAD, J., NEWLING, D.W. & TVETER, K. (1991). Prognostic factors in hormone-resistant progressing cancer of the prostate. *Annal. Oncol.* (in press).

HETHERINGTON, J.W., SIDDALL, J.K. & COOPER, E.H. (1988). Contribution of bone scintigraphy, prostatic acid phosphatase and prostate-specific antigen to the monitoring of prostate cancer. *Eur. Urol.*, **14**, 1–5.

ISAACS, J.T. (1984). The timing of androgen ablation therapy and/or chemotherapy in the treatment of prostatic cancer. *Prostate*, **5**, 1–17.

LEO, M.E., BILHARTZ, D.L., BERGSTRALH, E.J. & OESTERLING, J.E. (1991). Prostate specific antigen in hormonally treated stage D2 prostate cancer: is it always an accurate indicator of disease status? *J. Urol.*, **145**, 802–806.

MANNI, A., BARTHOLOMEW, M., CAPLAN, R., BOUCHER, A., SANTEN, R., LIPTON, A., HARVEY, H., SIMMONDS, M., WHITE-HERSHEY, D., GORDON, R., ROHNER, T., DRAGO, J., WETTLAUFER, J. & GLODE, L. (1988). Androgen priming and chemotherapy in advanced prostate cancer: evaluation of determinations of clinical outcome. *J. Clin. Oncol.*, **6**, 1456–1466.

MORGAN, W.R., ZINCKE, H., RAINWATER, L.M., MYERS, R.P. & KLEE, G.G. (1991). Prostate specific antigen values after radical retropubic prostatectomy for adenocarcinoma of the prostate: impact of adjuvant treatment (hormonal and radiation). *J. Urol.*, **145**, 319–323.

PAULSON, D.F., BERRY, W.R., COX, E.B., WALKER, A. & LASZLO, J. (1979). Treatment of metastatic endocrine-unresponsive carcinoma of the prostate gland with multiagent chemotherapy: indicators of response to therapy. *JNCI*, **63**, 615–622.

SOLOWAY, M.S., HARDEMAN, S.W., HICKEY, D., RAYMOND, J., TODD, B., SOLOWAY, S. & MOINUDDIN, M. (1988). Stratification of patients with metastatic prostate cancer based on extent of disease on initial bone scan. *Cancer*, **61**, 195–202.

STAMEY, T.A. & KABALIN, J.N. (1989). Prostate specific antigen in the diagnosis and treatment of adenocarcinoma of the prostate. I. Untreated patients. *J. Urol.*, **141**, 1070–1075.

STAMEY, T.A., KABALIN, J.N., MCNEAL, N.E., JOHNSTONE, J.M., FREIHA, F., REDWINE, E.A. & YANG, N. (1989*a*). Prostate specific antigen in the diagnosis and treatment of adenocarcinoma of the prostate. II. Radical prostatectomy treated patients. *J. Urol.*, **141**, 1076–1083.

STAMEY, T.A., KABALIN, J.N., FERRARI, M. & YANG, N. (1989*b*). Prostate specific antigen in the diagnosis and treatment of adenocarcinoma of the prostate. III. Radiation treated patients. *J. Urol.*, **141**, 1084–1087.

STAMEY, T.A., KABALIN, J.N., FERRARI, M. & YANG, N. (1989*c*). Prostate specific antigen in the diagnosis and treatment of adenocarcinoma of the prostate. IV. Anti-androgen treated patients. *J. Urol.*, **141**, 1088–1090.

WAEHRE, H., HOFF, WANDERAAS, E., PAUS, E. & FOSSÅ, S.D. (1991). Prediction of pelvic lymph node metastases by prostate specific antigen and prostatic acid phosphatase in clinical T3/T4 MO prostatic cancer. Submitted, 1992.